# Acute and Chronic Macrophage Differentiation Modulates TREM2 in a Personalized Alzheimer’s Patient-Derived Assay

**DOI:** 10.1007/s10571-023-01351-7

**Published:** 2023-05-17

**Authors:** Nicoleta-Carmen Cosma, Neriman Eren, Berk Üsekes, Susanna Gerike, Isabella Heuser, Oliver Peters, Julian Hellmann-Regen

**Affiliations:** Charité-Universitätsmedizin Berlin, corporate member of Freie Universität Berlin and Humboldt-Universität zu Berlin, Department of Psychiatry and Psychotherapy, Section Clinical Neurobiology, Charité – Universitätsmedizin Berlin, Hindenburgdamm 30, 12203 Berlin, Germany

**Keywords:** TREM2, LOAD, Monocyte-derived macrophages, Retinoic acid, Patient-derived personalized assay

## Abstract

Neuroinflammation plays a pivotal role in the pathogenesis of Alzheimer`s disease (AD). Brain macrophage populations differentially modulate the immune response to AD pathology according to the disease stage. Triggering receptor expressed on myeloid cells 2 (TREM2) is known to play a protective role in AD and has been postulated as a putative therapeutic target. Whether, and to which extent TREM2 expression can be modulated in the aged macrophage population of the brain is unknown, emphasizing the need for a human, patient-specific model. Using cells from AD patients and matched controls (CO) we designed an assay based on monocyte-derived macrophages to mimic brain-infiltrating macrophages and to assess the individualized TREM2 synthesis in vitro. We systematically assessed the effects of short-term (acute—2 days) and long-term (chronic—10 days) M1- (LPS), M2- (IL-10, IL-4, TGF-β), and M0- (vehicle) macrophage differentiation on TREM2 synthesis. Moreover, the effects of retinoic acid (RA), a putative TREM2 modulator, on individualized TREM2 synthesis were assessed. We report increased TREM2 synthesis after acute M2- compared to M1-differentiation in CO- but not AD-derived cells. Chronic M2- and M0-differentiation however resulted in an increase of TREM2 synthesis in both AD- and CO-derived cells while chronic M1-differentiation increased TREM2 in AD-derived cells only. Moreover, chronic M2- and M0-differentiation improved the amyloid-β (Aβ) uptake of the CO-derived whereas M1-differentiation of the AD-derived cells. Interestingly, RA-treatment did not modulate TREM2. In the age of personalized medicine, our individualized model could be used to screen for potential drug-mediated treatment responses in vitro.

**Graphical Abstract:**

Triggering receptor expressed on myeloid cells 2 (TREM2) has been postulated as a putative therapeutic target in Alzheimer’s disease (AD). Using cells from AD patients and matched controls (CO), we designed a monocyte-derived macrophages (Mo-MФs) assay to assess the individualized TREM2 synthesis in vitro. We report increased TREM2 synthesis after acute M2- compared to M1- macrophage differentiation in CO- but not AD-derived cells. Chronic M2- and M0- differentiation however resulted in an increase of TREM2 synthesis in both AD- and CO-derived cells while chronic M1-differentiation increased TREM2 in AD-cells only 
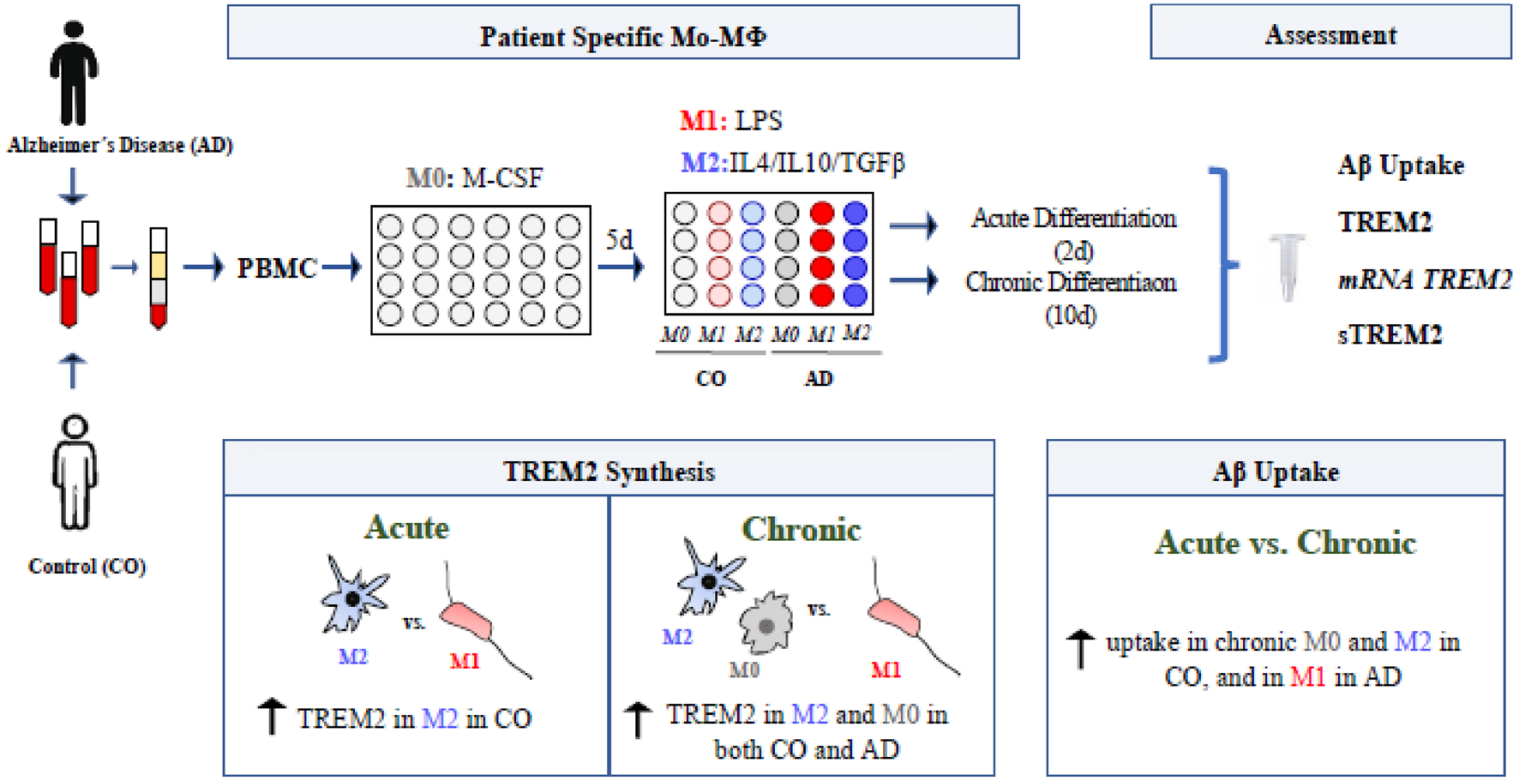

**Supplementary Information:**

The online version contains supplementary material available at 10.1007/s10571-023-01351-7.

## Introduction

Late-onset Alzheimer’s disease (LOAD) is the leading cause of dementia worldwide causing significant economic burden (Fleming et al., n.d.). With increasing life expectancy, the prevalence of Alzheimer’s disease (AD) is expected to rise, urging clinicians and researchers to find better treatment avenues. Converging evidence indicates that neuroinflammation modulates AD pathology (Bettcher et al. 2021; Regen, Hellmann-Regen, et al. 2017a, b) and points to a pivotal role of brain resident immune cells both in disease initiation and progression (Spangenberg et al. 2019; Villacampa and Heneka 2020). Most of the knowledge regarding immune cell function in AD focuses on microglia and stems from animal models of induced amyloid-β (Aβ) pathology, in which mutated genes from autosomal dominant AD are introduced into the mouse genetic background (Götz et al. 2004). The real-life patients’ biology especially in sporadic LOAD is however more complex. Up to 30–40 percent of patients have both AD pathology and vascular disease (Kapasi and Schneider 2016; Strickland 2018). This increases the likelihood of blood–brain barrier (BBB) dysfunction and might potentiate the contribution of peripherally derived myeloid cells to the neuroinflammatory processes seen in AD (Fiala et al. 1998; Garré and Yang 2018). Moreover, LOAD pathology occurs at later stages of life possibly involving immunosenescence, somatic mutations, and multimorbidity—factors related to aging and difficult to model in a transgenic mouse in which animals develop Aβ-pathology as early as 4 months (Yang et al. 2022). Thus, real-life patients` associated inflammatory pathology could significantly differ from animal studies (Drummond and Wisniewski 2017) and understanding the involvement of the aged innate immune cells in AD pathology at an individual patient’s level would require personalized patient-derived approaches.

Genome-wide association studies have identified several mutations of the triggering receptor expressed on myeloid cells 2 (TREM2) to be associated with AD (Jansen et al. 2019; Wightman et al. 2021). Converging evidence demonstrates that a TREM2-dependent mechanism is mandatory for developing a full protective damage-associated microglial (DAM) response to Aβ pathology (Deczkowska et al. 2018; Keren-Shaul et al. 2017; Krasemann et al. 2017; Mathys et al. 2017). A recent study showed that the DAM population detected in AD comprises TREM2-dependent microglia as well as monocyte-derived TREM2-expressing macrophages and that the later population also accumulates in the brain during physiological aging (Nevalainen et al. 2022; Silvin et al. 2022). These results are supported by studies showing increased soluble TREM2 (sTREM2) in the human cerebrospinal fluid (CSF) with aging and BBB breakdown (Heslegrave et al. 2016; Suárez‐Calvet et al. 2016). Moreover, increased sTREM2 was detected in the CSF of patients with mild cognitive impairment (MCI) due to AD but not AD dementia (Ewers et al. 2019; Heslegrave et al. 2016; Suárez‐Calvet et al. 2016). Prospective studies have suggested that apolipoprotein E4 (*APOE*ε*4*) carriers with increased sTREM2 exhibit slower disease progression compared to carriers with low sTREM2, supporting the theory of a protective effect (Edwin et al. 2020). Thus, the study of individualized TREM2 modulation in monocyte-derived macrophages (Mo-MФs) as a functional proxy for brain infiltrating macrophages in AD could offer insights into possible personalized therapeutic approaches (Cosma et al. 2021).

TREM2 is present not only on DAM but also on other organ-bound macrophages such as osteoclasts, lung, and peritoneal macrophages (Cella et al. 2003; Turnbull et al. 2006). In fact, several studies characterized TREM2 as a marker for infiltrating Mo-MФs (Molgora et al. 2020; Turnbull et al. 2006). More, TREM2 has been identified as an immunosuppressive marker in tumor-associated macrophages (Katzenelenbogen et al. 2020; Molgora et al. 2020). Since innate immune cells are highly adaptable (Gosselin et al. 2014), to assess the individual immune plasticity at a patient level, a form of standardization is necessary. Mo-MФs have been described according to their M1- and M2- macrophage differentiation profile, which although limited, has proven helpful in standardizing the assessment of innate immune plasticity (Cosma et al. 2021; Yang et al. 2014). In animal studies, TREM2 expression was shown to be induced by M-CSF, IL-4, and IL-13 stimulation of mouse macrophages in vitro and to be present in tumor-associated suppressive M2-like macrophages (Cella et al. 2003; Molgora et al. 2020; Turnbull et al. 2006). Whether this effect is also inducible in human Mo-MФs from aged healthy controls and AD patients is unknown. Moreover, since infiltrating macrophages can be long-lived at a tissue level, the effect of long-term (chronic) macrophage differentiation on TREM2 synthesis in AD-derived cells might be of therapeutic interest. Some animal studies found that retinoic acid receptor (RXR) agonists might increase TREM2 expression and that the promoter regions of the two genes are associated (Chen et al. 2020; Fitz et al. 2019; Savage et al. 2015). Retinoic acid (RA) stimulation was shown to increase the phagocytosis of myelin by macrophages and reduce the M1-polarization capacity (Wu et al. 2021). Whether RXR signaling modulates TREM2 in human patient-derived Mo-MФs is unknown.

To assess TREM2 synthesis at a functional and patient-specific level we developed an individualized Mo-MФs differentiation assay using peripheral blood cells from AD patients and matched controls. We examined the effect of acute (2 days, 1 × stimulation) and chronic (10 days, 3 × stimulations) M1-, M2-, and M0- macrophage differentiation on TREM2 synthesis in patient-derived individualized cell culture assays. Furthermore, we investigated the modulatory role of the putative anti-inflammatory and neuroprotective RA on TREM2 mRNA, secretion and protein synthesis in acute and chronic differentiated M1-, M2-, and M0- macrophages.

## Materials and Methods

### Participants

Patients with a confirmed clinical diagnosis of Alzheimer´s disease and healthy controls were enrolled. Controls were enrolled matched for age, APOE-status, and BMI (body mass index) (Table 1). All participants signed an informed consent form, and the local ethics committee approved the study (EA4/002/13). Individuals meeting the National Institute on Aging (NIA)—Alzheimer's Association Diagnostic Guidelines with a confirmed CSF diagnosis of AD according to the A + T + N + criteria were included (Calvin et al. 2020; Delmotte et al. 2021). Healthy controls, with confirmed A-T-N- criteria were recruited through the memory clinic of the department. All procedures complied with the ethical standards of national and institutional committees on human experimentation and with the Helsinki Declaration of 1975, as revised in 2008. All participants underwent a clinical interview, physical examination and their medical history was obtained. Exclusion criteria for all participants were any clinical signs of acute inflammation, recent infection requiring antibiotics (< 1 month), recent vaccination (< 3 months), chronic inflammatory disease, heart failure, clinically manifest asthma or allergies, history of cancer and stroke, pregnancy and lactation as well as poorly controlled diabetes, cardiovascular, renal, hepatic, hematologic, endocrine and neurologic disease, and any medication containing retinoic acid, chronic use of non-steroidal anti-inflammatory drugs or corticosteroids. Blood samples were taken between 8 and 12 a.m.

**Table 1:** Descriptive characteristic of AD patients and control subjects

	AD (*n* = 8)	CO (*n* = 8)	*p*-value
Age	74.25 (6.25)	72.75 (8.64)	0.9382
BMI (kg/m^2^)	23.52 (4.822)	22.46 (3.08)	0.7778
Gender (m/f)	4/4	2/6	> 0.9999
MMSE score	23.5 (1.4)	29.2 (1.03)	**0.0115**
APOE ε4 status (pos./neg.)	4/4	4/4	> 0.9999
CSF Aß 1–42	489.4 (248.3)	816.6 (402.1)	0.1049
CSF—p-tau_181_	112.5 (62.10)	38.31 (11.76)	**0.0007**
CSF—t-tau	654 (323.5)	252.6 (75.04)	**0.0002**
Disease duration (years)	2.21 (1.3)	n.a	

Significant *p*-values are given in bold

Data are shown as mean and standard deviation (SD) unless otherwise stated

Probability values (*p*) denote differences between groups. Kruskal–Wallis tests performed to compare gender and APOE ε4 differences. Pairwise comparison of groups was performed with Mann Whitney tests (unpaired groups)

*AD* Alzheimer´s disease; *CO* control, *APOE* apolipoprotein, *CSF* cerebrospinal fluid, *Aß* amyloid ß peptide, *P-tau*_*181*_ tau phosphorylated at threonine 181, *T-tau* total tau, *MMSE* Mini Mental State Examination, *n.a*. not applicable

### PBMC Isolation

Peripheral blood mononuclear cells (PBMCs) were obtained from heparinized venous blood, by using FICOLL™ density gradient centrifugation following previously published protocols (Cosma et al. 2021; Regen et al. 2017a, b). Anticoagulated blood was collected and then diluted twofold with PBS, pipetted into a centrifuge tube prefilled with Biocoll separating solution (Biochrom, Germany), followed by centrifuging at 2000 rpm for 30 min at room temperature without deceleration. Separated PBMC layers were washed twice with cold PBS and then stored at − 80 °C until further use.

### Primary Human Monocyte-Derived Macrophage Enriched Culture and Stimulation

**Fig. 1:**
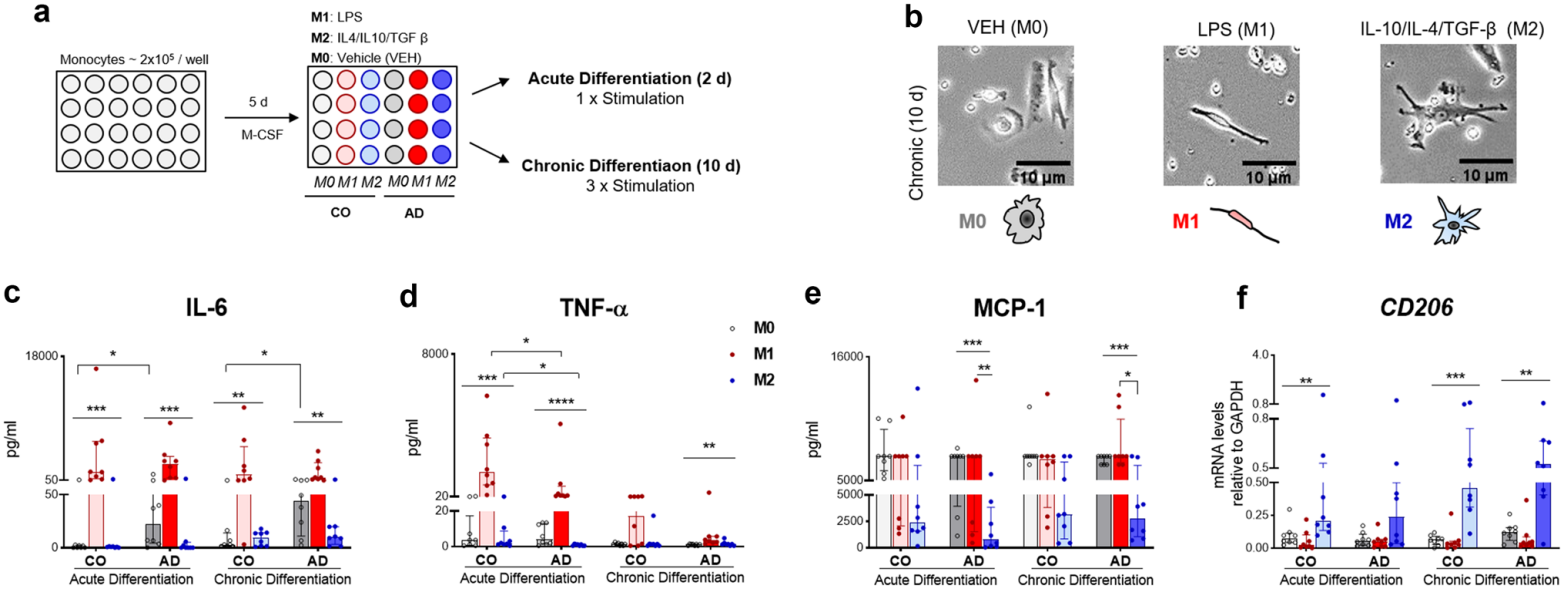
Acute and chronic Mo-MФs differentiation to M1-, M2-, and M0- macrophages **a** Experimental design: Mo-MФs were polarized for the total periods of 7 and 15 days with M-CSF, of which, respectively, the last 2 (acute) and 10 days (chronic) under differentiation to M1 (LPS), M2 (IL-4, IL10, TGF- β), and M0 (unstimulated) macrophages. **b** Morphology of the Mo-MФs after chronic differentiation observed with a Zeiss Axiovert 10 Inverted Microscope and captured with SWIFT Cam SC500 5.1 MP. Magnification = 25x, scale bar = 10 µm. **c** IL-6 and **d** TNF-α cytokine secretion; **e** MCP-1 secretion and (f) *CD206* mRNA expression levels in acute and chronic differentiated Mo-MФs from patients with AD (*n* = 8) and CO (*n* = 8). mRNA levels measured with RT-qPCR, normalized to *GAPDH*. Dots represent individual participant values. Closed bars and symbols represent M0 (light gray for CO; dark gray for AD), M1 (light red for CO; dark red for AD), and M2 (light blue for CO; dark blue for AD) macrophages, respectively. Friedman's ANOVA tests with Dunn's post hoc multiple comparison tests performed to analyze the within-group differences and pairwise comparison of groups was performed with Mann Whitney tests (unpaired groups) (*p < 0.05, **p < 0.01, ***p < 0.001)

Monocytes were isolated by adherence to plastic surfaces. For this, isolated PBMCs were cultured in a 24-well plate (2 × 10^5^ cells/well) at 37 °C with 9% O_2_ and 5% CO_2_ in RPMI medium enriched with 10% fetal calf serum (FCS, Biochrom, Germany), 1% Penicillin/Streptomycin (P/S) (10,000 U/10 mg per ml; Biochrom, Germany) and human macrophage -colony stimulating factor (M-CSF) (10 ng/ml) (Miltenyi Biotec). After overnight incubation, half of the RPMI-1640 media were exchanged for a high glucose-containing DMEM media supplemented with human M-CSF (10 ng/ml), 1% P/S and 10% FCS. Culture media were replaced with fresh DMEM media on day 2 of plating and all non-adherent cells were completely removed. Attached monocytes were polarized to macrophages (Mo-MФs) for a total period of 5 days with M-CSF, followed by 2 (acute, 1 × stimulation) and 10 days (chronic, 3 × stimulation) of differentiation to M1 (50 ng/ml LPS (Sigma-Aldrich, USA)), M2 (IL-4, IL-10, TGFβ each 20 ng/ml (Peprotech, USA)) according to previously published macrophage differentiation protocols (Cosma et al. 2021; Mia et al. 2014; Saeed et al. 2014) (Fig. 1a). See Supplemental Table 1 for cytokines and LPS lot numbers. Fresh medium change (25%) was performed at day 3 and 7 of differentiation. M-CSF-stimulated cells not incubated with any other stimuli were used as controls (unstimulated, M0-macrophages). To examine the RA effect, half of the cell culture plates of each participant were individually stimulated with RA (Retinoic acid, 2 µM; Sigma-Aldrich, USA, Fig. 3a). The RA was added to the wells 24 h after the M1-, M2-, and M0- differentiation. Each stimulation was run in duplicates, i.e., two wells per stimulation. All experiments were run simultaneously. See Figs. 1a and 3a for an overview of the stimulation protocol.

### Preparation of Cell Lysates

Mo-MФs were lysed with protein lysis buffer composed of 10 mM Tris pH 7.4, 150 mM NaCl, 2 mM EGTA, 50 mM ß-Glycerophosphate, 0.5% Triton X-100, 40 µl/ml 25 × cOmplete™ EDTA-free Protease Inhibitor Cocktail (Roche, Mannheim, Germany) and 50 µl/ml 20 × PhosSTOP™ (Sigma-Aldrich, Germany) after 2 (acute) and 10 days (chronic) macrophage differentiation. Cell lysates were stored at − 80 °C until measurement.

### Measuring Total Protein Concentration in Cell Lysates with BCA Assay

From each BCA standard (Thermo Scientific™ Pierce™ BCA protein assay reagents, MA, USA) and cell lysate sample, 10 µl were pipetted to each of the appropriate wells. BCA protein assay reagent B (Thermo Fisher Scientific Inc., MA, USA) was diluted with BCA protein assay reagent A (B: A = 1:50) (Thermo Fisher Scientific Inc., MA, USA) and 200 µl of the dilution was added to the standard and sample wells. 96-well plate was incubated for 1 h at RT and absorbance signals were read by using a multi-purpose Clariostar™ plate reader (BMG Labtech).

### Assessment of Neuroinflammatory Marker Levels

Secretion and cell-surface expression levels of proteins were measured in supernatants and cell lysates, respectively, by using the LEGENDplex Human Neuroinflammation Panel (13-plex: VILIP-1 (Visinin-like protein 1), MCP-1 (Monocyte chemoattractant protein-1), sTREM2 (soluble TREM2), BDNF (Brain-derived neurotrophic factor), TGF-β1 (Transforming growth factor beta), VEGF (Vascular endothelial growth factor), IL-6 (Interleukin-6), sTREM-1 (soluble TREM1), β-NGF (β-Nerve growth factor), IL-18 (Interleukin-18), TNF-α (Tumor necrosis factor-α), sRAGE (soluble receptor for advanced glycation end products), CX3CL1 (Fractalkine)) (BioLegend, San Diego, USA) as per the manufacturer’s instructions. Data were acquired using the BD FACS Canto™ II (Biosciences) and analysis was performed using LEGENDplex Data Analysis Software v8.0 (BioLegend, San Diego, USA) in accordance with the manufacturer’s instructions. Detection limit ranges for cytokines (pg/ml) are shown in supplemental Table 2. Measured cell-bound TREM2 protein levels in cell lysates were normalized to total protein concentrations assessed via BCA-Assay. All measurements were run in parallel, while the assay of AD and CO supernatants and cell lysates were performed on the same plate. CVs range was between 0.4 and 2.49%. VILIP-1, sTREM-1, β-NGF, IL-18, and CX3CL1 were measured under the limit of the detection for acute and chronic differentiated Mo-MФs.

### RNA Extraction and Quantitative Determination of mRNA Expression

Total RNA from Mo-MФs was isolated using TriReagent^R^ (Zymo-Research, Germany) according to manufacturers’ instructions, and extracted RNA was stored at − 80 °C for further analysis. Reverse transcription of the total RNA into cDNA was performed by following the standard protocol of the Revert Aid First Strand cDNA Synthesis Kit™ (Thermo Fisher Scientific Inc., MA, USA) and was stored at − 20 °C until use. The mRNA expression levels of *TREM2* and mannose receptor (Cluster of Differentiation 206, *CD206*) were quantified with the LightCycler™ 480 SYBR Green I Master (Roche, Mannheim, Germany) using 500 nM standard primer concentrations, following the manufacturer's instructions in an Applied Biosystems StepOne™ Real-Time PCR System (CA, USA). *GAPDH* was used as a housekeeping gene for data normalization (ΔCt) (Radonić et al. 2004). Relative quantification (ΔCt) and melting curve analysis were both carried out using the StepOne™ Real-Time PCR System software. The expression fold change was calculated as 2^−Δ*C*t^ and used for further analysis. Primer sequences are found in Supplemental Table 3.

### Uptake of Fluorescence-Aβ Peptide in Mo-MФ Cultures

Isolated PBMCs (10^5^ cells/well) were plated in 96 well black-clear bottom plates (Greiner Bio-One). Monocytes were isolated and polarized to M1 (LPS), M2 (IL-4, IL-10, TGF-β), and M0 (unstimulated) macrophages as indicated above. All experimental steps were performed with RPMI-1640 medium supplemented with 1% P/S, 10% FCS and M-CSF. To measure the phagocytic activity, macrophages were incubated with 500 nM HiLyte™ Fluor 488 Aβ_1‐42_ (Anaspec) for 4 h at 37 °C. Mo-MФs were washed twice with PBS and incubated with 1 μg/ml Hoechst dye 33,342 for 30 min at 37 °C. Extracellular fluorescence intensity of Aβ_42_ was quenched with 0.2% trypan blue in phosphate‐buffered saline (PBS) for 1 min. After aspiration, fluorescence signals were detected by using a multi-purpose Clariostar™ plate reader (BMG Labtech). Aβ uptake capacity of a cell was calculated as a ratio of total fluorescence intensity of HiLyte™ Fluor 488 Aβ_1‐42_ to Hoechst signal in per well and represented as the total Aβ_1‐42_ fluorescence signal within a cell.

### Data Analysis

Data analysis was performed using non-parametric statistics due to data distribution. GraphPad Prism version 8.0.2 (GraphPad Software, La Jolla, USA) was used for graphs and statistical analysis. Data are presented as median with interquartile range. Group differences were analyzed by Kruskal–Wallis tests with Dunn's post hoc multiple comparison test or Mann–Whitney U tests (unpaired; denoted by U) and Wilcoxon test (paired; denoted by W) for pairwise comparisons. Friedman's ANOVA tests with Dunn's post hoc multiple comparison test was performed to analyze the within-group differences. In all experiments, results were considered statistically significant when the *p* value of less than 0.05 was obtained. Cytokine levels below the detectable limit of the assay were included as half of the lower limit of detection (Cosma et al. 2021; Kiraly et al. 2017).

## Results

### Effective Acute and Chronic Mo-MФs Differentiation to M1- and M2- Macrophages

We recruited 16 clinically well-characterized participants including patients with AD (*n* = 8) and matched controls (*n* = 8). Patients and controls were subcategorized into *APOEε4* allele carrier vs. non carrier (n = 4 per group). Participants’ characteristics are shown in Table 1. After differentiating participant specific Mo-MФs for short-term (acute—2 days) and long-term (chronic -10 days) with LPS (M1), IL-4, IL-10, TGF-β (M2), or unstimulated (M0), respectively, we observed clearly distinguishable Mo-MФs´ morphologies: M1-macrophages were stretched and elongated cells while M2-macrophages had flat and enlarged amoeboid cell shapes and M0-macrophages showed roundish cell bodies (Fig. 1a and b).

We quantified IL-6 and TNF-α secretion (M1-marker) as well as *CD206* mRNA expression (M2-marker) to evaluate the M1-, respectively, M2-macrophage differentiation effectiveness (Fig. 1c–f). Friedman's ANOVA tests revealed a significant main effect of M1- and M2- differentiation in all the measured markers (Supplemental Table 4–5). AD-derived M0-macrophages showed higher IL-6 secretion in both acute and chronic differentiation compared to CO (U_M0/acute_ = 12, p_M0/acute_ = 0.0348; U_M0/chronic_ = 10, p _M0/chronic_ = 0.0191; Fig. 1c). TNF-α secretion was significantly increased in CO- compared to AD-cells in both acute M1- and M2-macrophages (U_M1/acute_ = 10, p_M1/acute_ = 0.019; U_M2/acute_ = 11.5, p_M2/acute_ = 0.0326; Fig. 1d). Furthermore, we evaluated the secretion levels of MCP-1 one of the important chemokines that regulate chemotaxis and infiltration of monocytes/macrophages to the site of inflammation and observed that AD-derived cells showed lower MCP-1 levels in M2 vs. M1 macrophages (pAD-M1vs.M2 = 0.008 in acute, pAD-M1vs.M2 = 0.012 in chronic; Fig. 1e; Supplemental Table 4–5).

### Impaired TREM2 Modulation After Acute Mo-MФs Differentiation in AD-Derived Cells

**Fig. 2:**
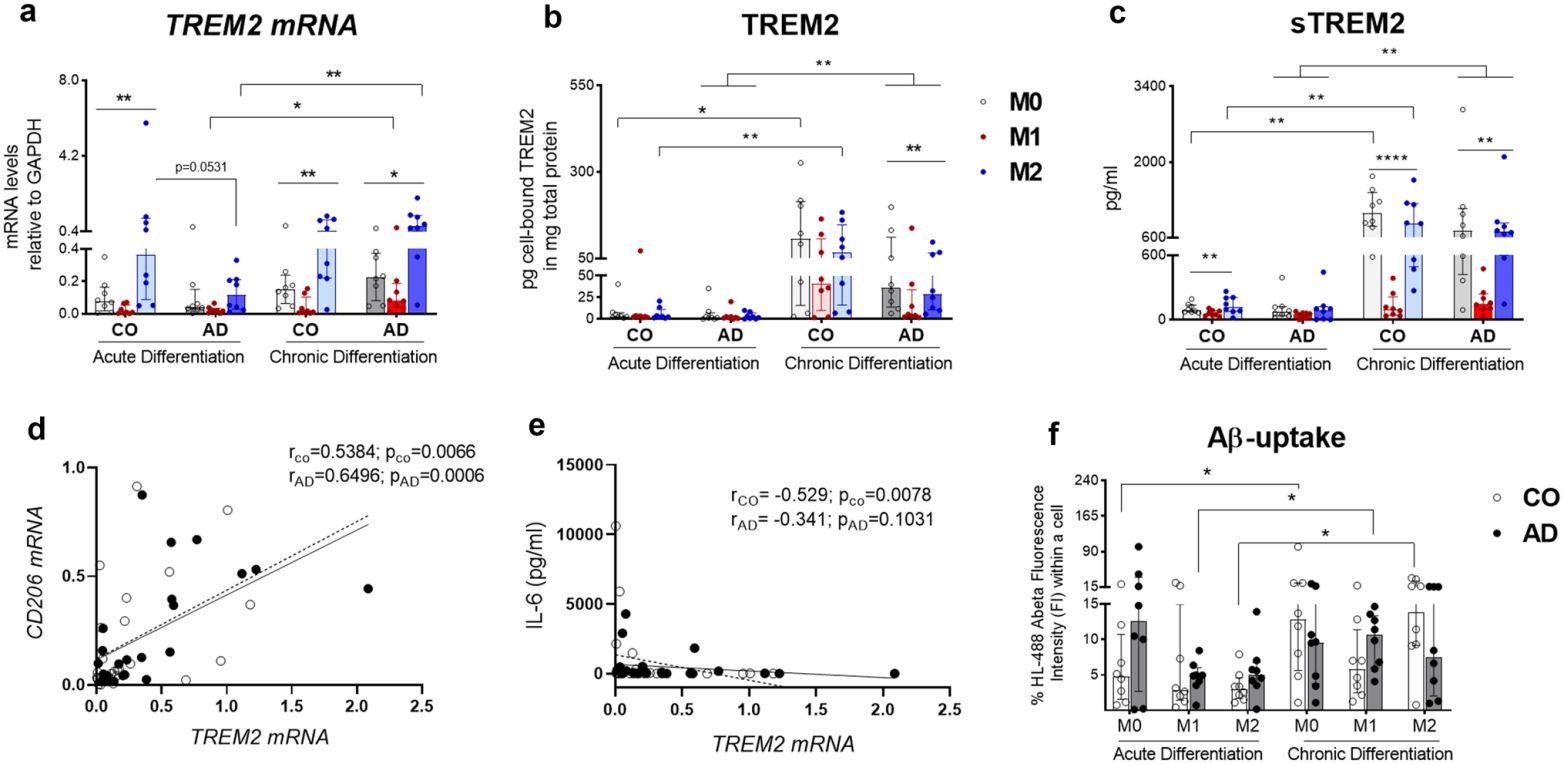
Impact of acute and chronic M1-, M2-, and M0- macrophage differentiation on TREM2 synthesis and Aβ uptake **a** *TREM2* mRNA, **b** cell-bound TREM2 and **c** sTREM2 levels in Mo-MФs cultures from AD patients (*n* = 8) and CO (*n* = 8). Correlation between *TREM2* mRNA expression and **d** *CD206* mRNA; and **e** IL-6 secretion in pooled Mo-MФs (including M0, M1, and M2) from AD patients (*n* = 24) and CO (*n* = 24) after chronic differentiation. Closed symbols with solid line represent AD while open symbols with line with dashes represent CO. The Spearman correlation test was used for correlation analysis where r represents the correlation coefficient. **f** Aβ-uptake by Mo-MФs from patients with AD (*n* = 8) and CO (*n* = 8). mRNA (normalization *GAPDH*) expression and protein secretion levels were measured with RT-qPCR and bead-based immunoassay, respectively. Cell-bound TREM2 protein levels are shown relative to total protein measured with BCA assay. Dots represent individual participant values. **a**–**c** Closed bars and symbols represent M0 (light gray for CO; dark gray for AD), M1 (light red for CO; dark red for AD), and M2 (light blue for CO; dark blue for AD) macrophages, respectively. **f** Open bars and symbols represent controls (CO) or closed bars and symbols AD (black). Friedman's ANOVA tests with Dunn's post hoc multiple comparison tests performed to analyze the within-group differences and pairwise comparison of groups was performed with Wilcoxon test (paired-) or Mann Whitney tests (unpaired groups) (*p < 0.05, **p < 0.01, ***p < 0.001)

To understand how M1-, M2-, or M0- differentiation modulates TREM2 in patient-derived Mo-MФs after acute (2 days) and chronic (10 days) differentiation, we assessed changes in mRNA (*TREM2*), protein (cell-bound TREM2), and secretion- (sTREM2) levels (Fig. 2a–c). Since 2 days in vitro stimulation of mouse macrophages with M-CSF and IL-4 was shown to induce TREM2 synthesis (Cella et al. 2003; Turnbull et al. 2006), we expected to see an increase of TREM2 at an individual level after M2 differentiation. After acute differentiation we indeed saw an increase in *TREM2* mRNA and sTREM2 in M2 compared to M1 only in CO-derived cells (Fig. 2a, c; Supplemental Table 5). In contrast, the AD-derived cells did not show an increase in *TREM2* mRNA expression or sTREM2 secretion after acute M2- differentiation (Fig. 2a, c; Supplemental Table 4). Interestingly the amount of cell-bound TREM2 remained constant after short-term differentiation in both groups regardless of differentiation status (Fig. 2b). More, CO- compared to AD-derived M2-macrophages showed higher *TREM2* mRNA expression (*U* = 13.50 *p* = 0.0531; Fig. 2a).

### Chronic Mo-MФs Differentiation Improves TREM2 Modulation in AD-Derived Cells

After chronic (10 days) differentiation however, both AD- and CO-derived cells revealed an increase in *TREM2* mRNA and sTREM2 in M2- compared to M1-diferentiation (Fig. 2a, c; Supplemental Table 4–5). More, the chronic M0- and M2- differentiated macrophages of both groups showed an increase in soluble and cell-bound TREM2 compared to the acute differentiation of the same participant-derived cells (Fig. 2b, c, Table 2). However, the chronic vs. acute M1-differentiation led to an increase in all measured levels of TREM2 only in AD- and not CO-derived cells (Fig. 2a–c, Table 2). These effects suggest that the AD-derived cells are capable of sustained TREM2 secretion if stimulated properly and for an adequate duration (Fig. 2c). Furthermore, it implies that TREM2 modulation in AD-derived cells is less sensitive to the chronic M1- “proinflammaory” deleterious effect. Moreover, after chronic differentiation *TREM2* mRNA levels positively correlated with M2-marker *CD206* in both AD-and CO-derived cells (Fig. 2d) but negatively correlated to M1 marker IL-6 only in CO-derived cells (Fig. 2e) further suggesting that TREM2 modulation in chronic AD- macrophages might be less sensitive to M1-differentiation.

**Table 2:** Impact of acute and chronic Mo-MФs differentiation on TREM2 synthesis

Gene*/Protein	M1							
	AD				CO			
	Acute (*n* = 8) Median (25%–75%)	Chronic (*n* = 8) Median (25%–75%)	Wilcoxon test	*p*-value	Acute (*n* = 8) Median (25%–75%)	Chronic (*n* = 8) Median (25%–75%)	Wilcoxon test	*p*-value
TREM2*	0.019 (0.01–0.03)	0.08 (0.04–0.12)	31	**0.031**	0.01 (0.003–0.055)	0.014 (0.01–0.104)	6	0.742
sTREM2	51.45 (15.15–65.3)	148.7 (101.1–239.8)	36	**0.008**	55.7 (38.79–85.89)	94.1 (41.6–213.7)	20	0.195
CB-TREM2	1.85 (0.82–2.86)	4.8 (2.74–34)	36	**0.008**	3 (2.04–4)	41.1 (10–106.5)	24	0.109

Gene*/Protein	M2 macrophages							
	AD				CO			
	Acute (*n* = 8) Median (25%–75%)	Chronic (*n* = 8) Median (25%–75%)	Wilcoxon test	*p*-value	Acute (*n* = 8) Median (25%–75%)	Chronic (*n* = 8) Median (25%–75%)	Wilcoxon test	*p*-value
TREM2*	0.17 (0.028–0.21)	0.67 (0.4–1.2)	36	**0.008**	0.363 (0.09–1.1)	0.43 (0.22–1)	5	0.77
sTREM2	91.25 (3.35–123)	712.6 (598.5–873)	36	**0.008**	121.2 (78–207)	886.1 (492–1233)	36	**0.008**
CB-TREM2	2.24 (0.33–7.1)	28.8 (10.8–65.5)	36	**0.008**	2.63 (1.55–11.2)	67.5 (16.4–147)	36	**0.008**

Gene*/Protein	M0 macrophages							
	AD				CO			
	Acute (*n* = 8) Median (25%–75%)	Chronic (*n* = 8) Median (25%–75%)	Wilcoxon test	*p*-value	Acute (*n* = 8) Median (25%–75%)	Chronic (*n* = 8) Median (25%–75%)	Wilcoxon test	*p*-value
TREM2*	0.04 (0.03–0.15)	0.224 (0.08–0.374)	14	0.382	0.78 (0.02–0.164)	0.15 (0.06–0.24)	18	0.234
sTREM2	73.84 (36.7–123)	730 (421–1137)	36	**0.008**	89 (72.3–138.5)	1051 (812–435)	36	**0.008**
CB-TREM2	1.7 (0.42–7.14)	36.13 (14–111)	36	**0.008**	3.1 (2.2–7.2)	107 (15.7–213.4)	34	**0.016**

Significant *p*-values are given in bold

*qPCR products in italic

TREM2* triggering receptor expressed on myeloid cells 2, *sTREM2* soluble triggering receptor expressed on myeloid cells 2, *CB-TREM2* cell bound triggering receptor expressed on myeloid cells 2

### Chronic Mo-MФs Differentiation Modulates Aβ Uptake

Since TREM2 has been shown to modulate the accumulation of Aβ species in extracellular plaques (Joshi et al. 2021), we wondered whether the differentiation-induced modulation of TREM2 might also affect the phagocytosis capacity of the Mo-MФs. We observed that chronic macrophage differentiation resulted in significantly higher Aβ uptake compared to acute differentiation both in M2- and M0- macrophages of CO-derived (W_M2_ = 34, *p* = 0.0156) (W_M0_ = 32, *p* = 0.0234; Fig. 2f). However, AD-derived cells showed significantly more Aβ uptake over time in M1-macrophages (*W* = 30, *p* = 0.0391, Mdn_acute_ = 4.93, Mdn_chronic_ = 10.65; Fig. 2f). We however did not find a difference in Aβ uptake between AD- and CO-derived cells (Fig. 2f).

### RA Treatment Does Not Modulate TREM2 in Acute and Chronic Mo-MФs Cultures

**Fig. 3:**
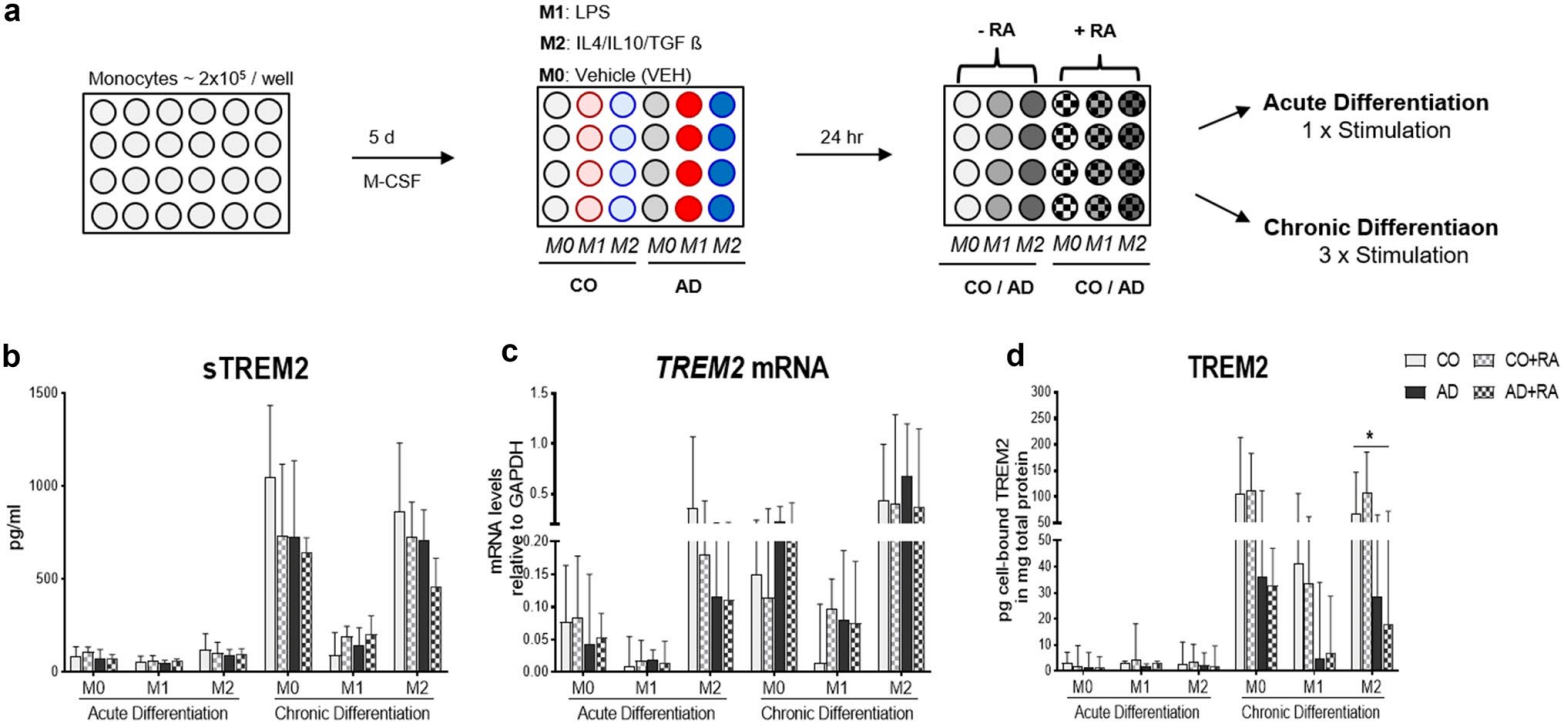
Retinoic acid (RA) does not modulate TREM2 synthesis in response to the acute and chronic Mo-MФs differentiation **a** RA stimulation performed 24 h after Mo-MФs differentiation to M1 (LPS), M2 (IL-4, IL10, TGF- β), or M0 (VEH, unstimulated) macrophages. RA-stimulation effect on **b** sTREM2, **c** *TREM2* mRNA and **d** cell-bound TREM2 protein levels in Mo-MФs cultures from AD (*n* = 8) and CO (*n* = 8)-derived cells in acute and chronic differentiation. Cell-bound TREM2 protein levels shown relative to total protein measured with BCA assay. Dots represent individual participant values. Closed bars without pattern represent CO (gray) and AD (black) and open bars with pattern represent CO + RA (gray) and AD + RA (black). The most relevant statistically significant differences between groups are shown by Kruskal–Wallis tests with Dunn's post hoc multiple comparison tests (*p < 0.05, **p < 0.01, ***p < 0.001)

To assess one potential pharmacologic treatment strategy, we investigated whether RA-stimulation additionally modulates TREM2 synthesis in acute and chronic patient-derived M0-, M1-, and M2-Mo-MФ cultures. Figure 3 shows measured-TREM2 levels after additional RA-treatment. We did not find an additional RA-dependent modulation of TREM2 levels in acute or chronic patient-derived M0-, M1-, and M2-differentiated macrophages (Fig. 3b–d).

### Higher TREM2 Secretion in APOEε4(+) Compared to APOEε4(−) Mo-MФs

**Fig. 4:**
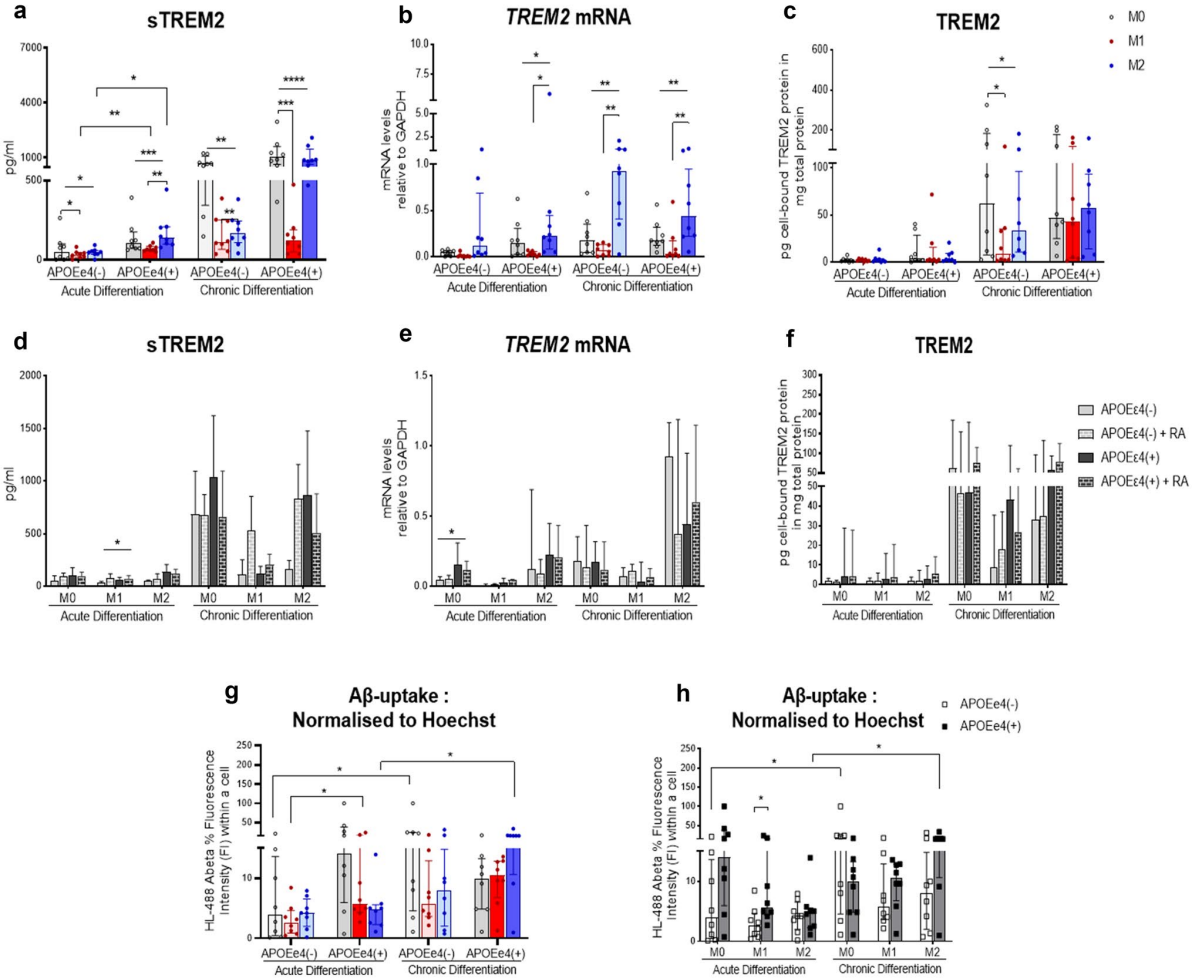
APOEε4 effect on TREM2 synthesis in the absence or presence of RA-stimulation **a** sTREM2, **b** *TREM2* mRNA and **c** cell-bound TREM2 protein levels in Mo-MФs cultures from APOEε4(+) (*n* = 8) and APOEε4(−) (*n* = 8)- derived cells. Dots represent individual participant values. Closed bars and symbols represent M0 (light gray for APOEε4(−); dark gray for APOEε4(+)), M1 (light red for APOEε4(−); dark red for APOEε4(+)), and M2 (light blue for APOEε4(-); dark blue for APOEε4(+)) macrophages, respectively. RA-stimulation effect on **d** sTREM2, **e** *TREM2* mRNA and **f** cell-bound TREM2 protein levels in Mo-MФs cultures after acute and chronic differentiation. Closed bars without pattern represent APOEε4(−) (gray) and APOEε4(+) (black) and open bars with pattern represent APOEε4(−) + RA (gray) and APOEε4(+) + RA (black). **g** and **h** Measurement of Aß-uptake by Mo-MФs from patients with APOEε4(+) (*n* = 8) and APOEε4(−) (*n* = 8). Fluorescence intensity in Mo-MФs cultures monitored with a microplate reader. mRNA (normalization *GAPDH*) expression and protein/secretion levels were measured with RT-qPCR and bead-based immunoassay, respectively. Cell-bound TREM2 protein levels are shown relative to total protein measured with the BCA assay. For the group comparison, open bars and symbols represent controls APOEε4 (−) or closed bars and symbols APOEε4 (+) (black). Friedman's ANOVA tests were performed to analyze the within-group differences, while Kruskal–Wallis tests with Dunn's post hoc multiple comparison tests were used to analyze differences between groups. Pairwise comparison of groups was performed with Wilcoxon test (paired-) or Mann Whitney tests (unpaired groups) (*p < 0.05, **p < 0.01, ***p < 0.001)

We further evaluated whether TREM2 synthesis may be differentially regulated by APOEε4 genotype in acute and chronic patient-derived Mo-MФs (Fig. 4a–c). We found that acute M1- and M2- differentiated macrophages secreted significantly higher sTREM2 in APOEε4(+) compared to APOEε4(−) (U_M1/acute_ = 6, p_M1/acute_ = 0.0042; U_M2/acute_ = 10.5, p_M2/acute_ = 0.0.025; Fig. 4a). However, APOEε4(+) compared to APOEε4(−) derived Mo-MФs did not show different *TREM2* mRNA and cell-bound protein expression levels (Fig. 4b, c). More, there was also no interaction between retinoid treatment and APOE genotype (Fig. 4d–f). Furthermore, we evaluated whether the APOEε4 genotype mediates Aβ uptake in acute and chronic Mo-MФs cultures. M1-macrophages from APOEε4(+) showed higher Aβ uptake compared to the APOEε4(−) (*U *= 10, *p* = 0.0207, Mdn_ApoEε4(+)_ = 5.680, Mdn_ApoEε4(−)_ = 2.610; Fig. 4h).

We further assessed whether APOEε4 genotype would modulate acute and chronic Mo-MФs differentiation and did not see any genotype-specific effect on the synthesis of M1- and M2- marker as well as other macrophages secreted cytokines (MCP-1, VEGF, and sRAGE) (Supplemental Fig. 1 ; for statistics Supplemental Table 6–7).

## Discussion

In this work, we investigated the potential and dynamics of TREM2 modulation by acute and chronic M0-, M1-, and M2- macrophage differentiation in an individualized patient-specific assay comparing Mo-MФs from well-characterized LOAD patients with controls. Moreover, we assessed whether RA-stimulation might additionally modulate TREM2 synthesis. We show differences in *TREM2* mRNA and sTREM2 levels after acute M2- compared to M1- macrophage differentiation only in CO- and not AD-derived cells. Moreover, we report a significant increase in sTREM2 and cell-bound TREM2 after chronic vs. acute Mo-MФs differentiation both in CO- and AD-derived cells in M0- and M2-differentiation. Chronic vs. acute M1-differentiation however increased TREM2 only in AD-derived cells.

First, we observed robust M0-, M1-, M2- differentiation patterns in AD- and CO- derived cells, both at the morphological level as well as through specific markers in line with previous published studies (Cosma et al. 2021; Ormel et al. 2017). As expected, acute and chronic M1 vs. M2 differentiated macrophages, respectively, showed upregulated specific pro- vs. anti-inflammatory markers in both groups, demonstrating that our patient-derived individualized cell culture assay is a proper in vitro stimulation system to emulate training and tolerance of tissue macrophages at a patient-specific level. Furthermore, we found significantly higher IL-6 secretion in AD compared to CO after acute- and chronic M0-macrophage differentiation, which is consistent with studies that show higher IL-6 levels in AD patients (Licastro et al. 2000; Saresella et al. 2020).

TREM2 has been proposed to play a modulatory role in the progression of AD (Ulrich et al. 2017). Contrasting data from AD patients report both a reduction and an increase of TREM2 in the myeloid cells of the central and peripheral compartments, respectively (Casati et al. 2018; Lue et al. 2015). At the transcript level, we observed higher *TREM2* mRNA levels in CO compared to AD in short-term differentiated M2-macrophages. Since our design was focused on mimicking differentiated brain infiltrating macrophages and not peripheral blood cells, our results would match the above-mentioned reports (Casati et al. 2018; Hu et al. 2014; Lue et al. 2015). Moreover, *TREM2* mRNA was positively correlated with *CD206* mRNA levels in both groups and negatively correlated with pro-inflammatory IL-6 in CO-cells. These results are in the line with other studies suggesting that type II immune response induces TREM2 synthesis (Liu et al. 2020; Ydens et al. 2012).

Preliminary clinical studies revealed increased sTREM2 levels associated with microglial activation along with AD progression (Franzmeier et al. 2020; Heslegrave et al. 2016; Suárez‐Calvet et al. 2016). Our data suggest M2- but also M0- macrophages are a main source of sTREM2 in both AD- and CO-derived cells. To our knowledge, this is the first study to show increased TREM2 after chronic (10 days) M2- and M0- macrophage differentiation, suggesting TREM2 as a marker for native (M0) or anti-inflammatory (M2) differentiated macrophages. Interestingly aged AD-derived cells show the same secretion potential as CO after the chronic differentiation, suggesting that M0- or M2- modulatory substances could have a therapeutic potential in AD. Both acute and chronic M1-differentiation suppressed TREM2 synthesis in both groups in accordance with other reports (Colonna 2023). However, chronic vs. acute M1-differentiation seemed to suppress TREM2 less in AD- than CO-derived cells. Together the lack of negative correlation with IL-6 seen in chronic differentiated AD-derived cells, this might suggest that TREM2 modulation in chronic differentiated Mo-MФs form AD-patients might be less sensitive to M1-differentiation than CO-derived cells.

The increase in cell-bound and soluble TREM2 after M0- as well as M2- differentiation implicates this molecule in tissue repair mechanisms. Our results are thus in line with research looking at TREM2 function in other tissues like lung macrophages or tumor-associated macrophages (Byers et al. 2018; Katzenelenbogen et al. 2020; Molgora et al. 2020; Nakamura & Smyth 2020). Murine studies showed increased TREM2 synthesis in response to acute stimulation with M-CSF and IL-4 in vitro and the first human studies noted that the population of M2-macrophages is decreased in AD (Saresella et al. 2020; Turnbull et al. 2006). However, most of these studies focused on short-term differentiation (hours up to 2 days). We thus advance the field by showing that AD-derived cells are capable of sustained TREM2 synthesis at a comparable level with CO if long-term stimulated and suggest that longer (ex. 10 days) macrophage differentiation assays should be considered for future characterization of patient-derived in vitro assays.

*APOEε4* variant has been identified as the most common genetic risk factor for AD and proven to be a ligand for TREM2, suggesting that the interplay between APOE-TREM2 might play a role in the regulation of cellular functions (Jendresen et al. 2017; Shi & Holtzman 2018; Wolfe et al. 2018). We found a tendency toward higher *TREM2* mRNA expression in APOEε4 carriers vs. non-carriers after acute M1-macrophage differentiation, which is consistent with some studies of AD patients (Casati et al. 2018; Mori et al. 2015). We report increased sTREM2 levels in acute differentiated M1- and M2-macrophages in APOEε4(+) relative to APOEε4(−). Franzmeier et al. have shown that APOEε4 carriers with elevated sTREM2 levels present slower cognitive decline and neurodegeneration compared to carriers with low levels of sTREM2 (Franzmeier et al. 2020), further suggesting sTREM2 as a promising therapeutic target, especially in APOEε4 carriers.

Retinoid signaling regulates the expression of various genes involved in AD pathogenesis via activating transcriptional processes through RA-binding receptors including retinoic acid receptors and retinoid X receptors (Goodman & Pardee 2003; Lee et al. 2009). One recent study showed that two-day all-trans retinoic acid (atRA) plus IL-4 in vitro treatment of THP-1 macrophages significantly promotes *TREM2* mRNA expression (Chen et al. 2020). Contrary to these results, we did not observe significant additional effects of RA treatment on TREM2 synthesis in our patient-derived Mo-MФs cultures. Differences in stimulation conditions (IL-4 vs. IL-4/IL-10/TBF-β in our study), cell cultures (THP-1 vs. participant-derived in our study) as well as number of separate assays (3 per group vs. 8 per group in our study), may account for the different results. Moreover, while Chen et al. assessed *TREM2* mRNA only, our assessment of TREM2 at several levels of production revealed no additional RA-induced effect regardless of differentiation phenotype. However, future studies looking into RA-dependent modulation of TREM2 might consider stimulation at other concentrations than used in our study.

The small number of participants constitutes a limitation of the study. Our study was intended to assess for the first time the suitability of TREM2 modulation in a personalized human in vitro cell model. This study was not powered to observe small effect sizes between AD- and CO-derived cells that would likely not significantly add to the diagnostic precision of established biomarkers. However, by running all the experiments in parallel we indented to minimize other confounding factors potentially influencing the individualized, cell-based assay. The dichotomous M1- and M2- macrophage differentiation theory is heavily debated since advances in single-cell technologies clearly suggest that both macrophages and microglia have a complex response to immune stimuli and usually coexist at the same tissue target (Silvin et al. 2022). However, this technique has proven useful in standardizing the assessment of innate immune plasticity especially when stimulation of patient-derived cells at an individual level is assessed (Cosma et al. 2021; Yang et al. 2014). Moreover, the goal of our proof concept study was to assess whether human, aged Mo-MФs show effective TREM2 modulation in response to stimuli otherwise well described in animal models (Katzenelenbogen et al. 2020; Turnbull et al. 2006).

## Conclusion

This is the first study to assess TREM2 dynamics in patient-specific long-term macrophage differentiation assay. We report a significant increase in sTREM2 and cell-bound TREM2 after chronic Mo-MФs differentiation both in CO- and AD- derived cells, especially after M2-differentiation. Since M2-macrophages are thought to be neuroprotective, we speculate that shifting the macrophage phenotype toward an alternative activation state may significantly increase TREM2 synthesis in AD patients and thus present a potential therapeutic avenue. Finally, we point out that patient-derived individualized cell culture assays may offer a chance to develop or screen for novel therapeutic strategies in AD in a precision-medicine approach. However, adequate stimulation duration needs to be considered for in vitro assays especially when long-term effects are sought.

## Supplementary Information

Below is the link to the electronic supplementary material.

APOEε4 does not regulate the acute and chronic M1-, M2- and M0- macrophage differentiation (a) MCP-1, (b) VEGF (c) sRAGE, (d) IL-6, (e) TNF-α and (f) *CD206* mRNA levels in Mo-MФs cultures from APOEε4 (+) (n=8) and APOEε4 (-) (n=8)-derived cells. Dots represent individual participant values. Closed bars and symbols represent M0 (light grey for CO; dark grey for AD), M1 (light red for APOEε4(-); dark red for APOEε4(+) ) and M2 (light blue for APOEε4(-); dark blue for APOEε4(+)) macrophages respectively. Friedman's ANOVA tests performed to analyze the within-group differences (*p<0.05, **p<0.01, ***p<0.001). Supplementary file1 (TIF 204 kb)

Gender or APOEε4 genotype does not regulate TREM2 synthesis in AD (a) sTREM2, (b) TREM2 mRNA and (c) cell-bound TREM2 protein levels in Mo-MФs cultures from AD-APOEε4(+) (n=4) and AD-APOEε4(-) (n=4)- derived cells. (d) sTREM2, (e) TREM2 mRNA and (f) cell-bound TREM2 protein levels in Mo-MФs cultures from AD-female(+) (n=4) and AD-male(-) (n=4)- derived cells. Dots represent individual participant values. Pairwise comparisons of groups were performed with the Wilcoxon test (paired) (*p<0.05, **p<0.01, ***p<0.001). Supplementary file2 (TIF 221 kb)

Supplementary file3 (DOCX 46 kb)

## Data Availability

All relevant data in this study are available upon reasonable request directed to the corresponding author.

## Abbreviations

AD: Alzheimer’s disease
TREM2: Triggering receptor expressed on myeloid cells 2
CO: Control
Mo-MФs: Monocyte-derived macrophages
RA: Retinoic acid
Aβ: Amyloid-β
LPS: Lipopolysaccharide
IL-4: Interleukin-4
IL-10: Interleukin-10
TGF-β: Transforming growth factor beta
LOAD: Late-onset Alzheimer’s disease
APOE: Apolipoprotein E
BBB: Blood–brain barrier
MCI: Mild cognitive impairment
sTREM2: Soluble TREM2
CSF: Cerebrospinal fluid
PD: Parkinson’s disease
RXR: Retinoic acid receptor
NIA: National Institute on Aging
PBMC: Peripheral blood mononuclear cells
FCS: Fetal calf serum
P/S: Penicillin/streptomycin
M-CSF: Macrophage-colony stimulating factor
BCA: Bicinchoninic acid
VILIP-1: Visinin-like protein 1
MCP-1: Monocyte chemoattractant protein-1
sTREM-1: Soluble TREM1
BDNF: Brain-derived neurotrophic factor
TGF-β1: Transforming growth factor beta 1
VEGF: Vascular endothelial growth factor
IL-6: Interleukin-6
TNF-α: Tumor necrosis factor-α
β-NGF: β-Nerve growth factor
IL-18: Interleukin-18
sRAGE: Soluble receptor for advanced glycation end products
CX3CL1: Fractalkine
CD206: Cluster of differentiation 206
GAPDH: Glyceraldehyde-3-phosphate dehydrogenase
Δ*C*_t_: Data normalization relative to housekeeping gene
PBS: Phosphate‐buffered saline
SEM: Standard error of the mean
atRA: All-trans retinoic acid
