# Supplementary figures and images for: Acute and Chronic Macrophage Differentiation Modulates TREM2 in a Personalized Alzheimer’s Patient-Derived Assay
Source: Cell Mol Neurobiol. 2023 May 17;43(6):3047–60. doi: 10.1007/s10571-023-01351-7 (PMC10333375; doi:10.1007/s10571-023-01351-7)

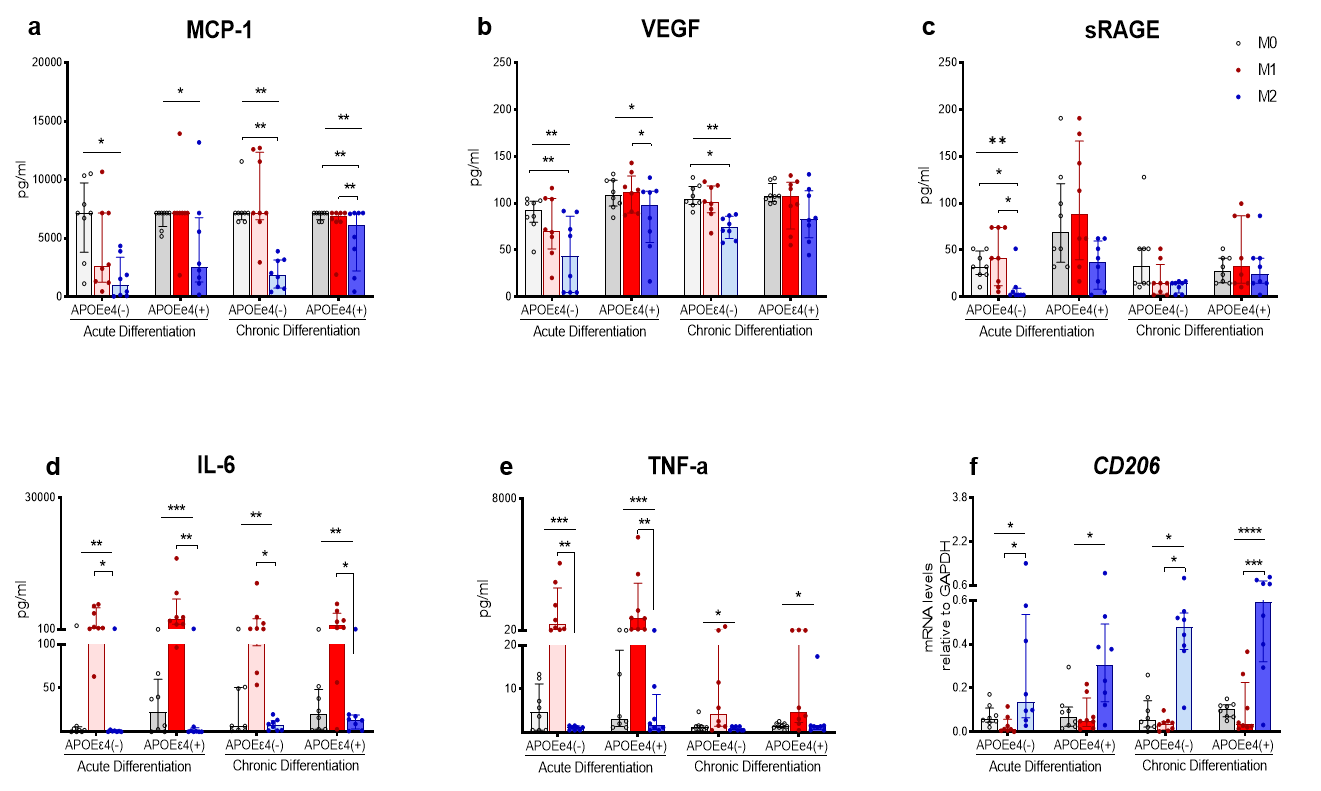

Supplement: Supplementary file 1 — APOEε4 does not regulate the acute and chronic M1-, M2- and M0- macrophage differentiation (a) MCP-1, (b) VEGF (c) sRAGE, (d) IL-6, (e) TNF-α and (f) CD206 mRNA levels in Mo-MФs cultures from APOEε4 (+) (n=8) and APOEε4 (-) (n=8)-derived cells. Dots represent individual participant values. Closed bars and symbols represent M0 (light grey for CO; dark grey for AD), M1 (light red for APOEε4(-); dark red for APOEε4(+) ) and M2 (light blue for APOEε4(-); dark blue for APOEε4(+)) macrophages respectively. Friedman's ANOVA tests performed to analyze the within-group differences (*p<0.05, **p<0.01, ***p<0.001). Supplementary file1 (TIF 204 kb) [file 10571_2023_1351_MOESM1_ESM.tif]

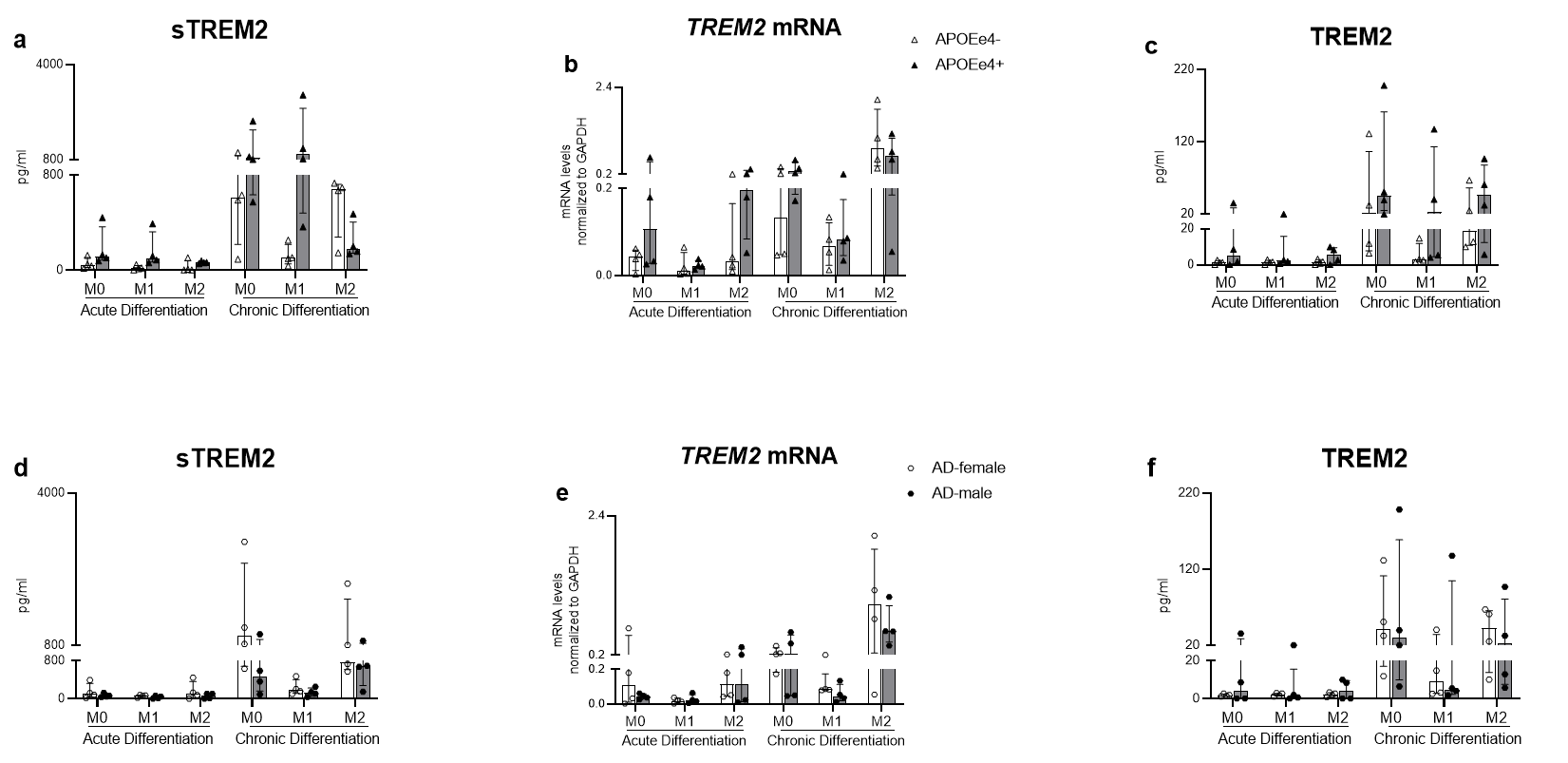

Supplement: Supplementary file 2 — Gender or APOEε4 genotype does not regulate TREM2 synthesis in AD (a) sTREM2, (b) TREM2 mRNA and (c) cell-bound TREM2 protein levels in Mo-MФs cultures from AD-APOEε4(+) (n=4) and AD-APOEε4(-) (n=4)- derived cells. (d) sTREM2, (e) TREM2 mRNA and (f) cell-bound TREM2 protein levels in Mo-MФs cultures from AD-female(+) (n=4) and AD-male(-) (n=4)- derived cells. Dots represent individual participant values. Pairwise comparisons of groups were performed with the Wilcoxon test (paired) (*p<0.05, **p<0.01, ***p<0.001). Supplementary file2 (TIF 221 kb) [file 10571_2023_1351_MOESM2_ESM.tif]
