# Supplementary material for: Acute and Chronic Macrophage Differentiation Modulates TREM2 in a Personalized Alzheimer’s Patient-Derived Assay
Source: Cell Mol Neurobiol. 2023 May 17;43(6):3047–60. doi: 10.1007/s10571-023-01351-7 (PMC10333375; doi:10.1007/s10571-023-01351-7)
Supplement: Supplementary file 3 — Supplementary file3 (DOCX 46 kb) [file 10571_2023_1351_MOESM3_ESM.docx]

| Supplemental Table 1. Cytokines used for differentiation | | |
| --- | --- | --- |
| source | **Macrophage Differentiation** | **Inflammatory Differentiation** |
| *Human* | M1- Macrophage | 50 ng/ml LPS; L6529 |
|  | M2-Macrophage | 20 ng/ml IL-4; Lot 091514  20 ng/ml IL-10; Lot 110621  20 ng/ml TGF β ; Lot 0506S354 |
|  | M0- Macrophage | 10 ng/ml M-CSF |
| *LPS- Lipopolysaccharide; IL-4-Interleukin-4; IL-10-Interleukin-10 ; TGFß -Transforming growth factor beta; M-CSF-Macrophage colony-stimulating factor* | | |

| **Supplemental Table 2. Cytokine detection limit range (pg/ml)** | |  |
| --- | --- | --- |
|  | **Acute Differentiation** | **Chronic Differentiation** |
| sTREM2 | 1.87 - 567.12 | 36.36 - 2973.8 |
| CB-TREM2 | 0.093 – 71.693 | 1.528 – 326.09 |
| IL-6 | 0.215 – 16225 | 0.215 – 16427 |
| TNF-α | 0.605 - 56658 | 0.605 – 252,92 |
| MCP-1 | 47.53 - 13947 | 430.44 - 12786 |
| *TREM2* – triggering receptor expressed on myeloid cells 2; *sTREM2*- soluble triggering receptor expressed on myeloid cells 2; CB -TREM2 -cell bound triggering receptor expressed on myeloid cells 2; *IL-6* -interleukin 6; *TNFα*- tumor necrosis factor α; *MCP-1* - monocyte chemoattractant protein-1. | | |

| Supplemental Table 3. Primer sequence (5’ > 3’) | | |
| --- | --- | --- |
| *trem2* | F: CAG CCA TCA CAG ACG ATA CCC | R: AAG TGG GTG GGA AGG GGA TTT C |
| *GAPDH* | F: TTG CCA TCA ATG ACC CCT TCA | R: CGC CCC ACT TGA TTT TGG A |
| *CD 206* | F: TGC AGA AGC AAA CCA AAC CTG TAA | R: CAG GCC TTA AGC CAA CGA AAC T |
| *TREM2 – Triggering receptor expressed on myeloid cells 2; GAPDH- Glyceraldehyde-3-phosphate dehydrogenase; CD206 - Cluster of differentiation 206 (mannose receptor);* | | |

| Supplemental Table 4. Acute and chronic differentiation effect Mo-Mф from AD- derived cells. | | | | | | | | | | |  |
| --- | --- | --- | --- | --- | --- | --- | --- | --- | --- | --- | --- |
|  | **AD** | | | | | | | | | |  |
| *Gene**/  Protein | **Acute Differentiation** | | | | | **Chronic Differentiation** | | | | |  |
|  | Friedman statistic | p-value | Dunn´s multiple comparison tests | | | Friedman statistic | p-value | Dunn´s multiple comparison tests | | |  |
|  |  |  | M1.vs M2 | M1.vs M0 | M2.vs M0 |  |  | M1.vs M2 | M1.vs M0 | M2.vs M0 |  |
| *trem2* | | 4.467 | **0.0108** | 0.1365 | 0.5074 | >0.9999 | 7.750 | **0.0179** | **0.0179** | 0.9519 | 0.2404 |
| sTREM2 | | 4.323 | 0.1177 | 0.5074 | 0.1365 | >0.9999 | 12 | **0.0011** | **0.00081** | **0.00081** | >0.9999 |
| CB-TREM2 | | 0.25 | 0.9674 | >0.9999 | >0.9999 | >0.9999 | 10.75 | **0.0024** | 0.1365 | **0.0035** | 0.6339 |
| *cd206* | | 4 | 0.1495 | 0.1365 | 0.9519 | 0.9516 | 9 | **0.0099** | **0.0081** | 0.4008 | 0.4008 |
| IL-6 | | 13.61 | **0.0001** | **0.0009** | 0.3125 | 0.1365 | 9.750 | **0.0048** | **0.0081** | **0.0733** | >0.9999 |
| TNF- α | | 15.55 | **<0.0001** | **0.0003** | 0.1008 | 0.2404 | 9 | **0.0064** | **0.0260** | 0.1824 | >0.9999 |
| mcp-1 | | 13.71 | **0.0005** | **0.0081** | >0.9999 | 0.0081 | 13.13 | **0.0007** | **0.0121** | >0.9999 | 0.0526 |
| *qPCR products* (in italic)* and proteins calculated using Friedman’s ANOVA with Dunn´s multiple comparison tests for all measured markers in M1, M2, and M0 after acute and chronic differentiation. *TREM2 –* triggering receptor expressed on myeloid cells 2*; CD206 - cluster of differentiation 206 (mannose receptor);* sTREM2- soluble triggering receptor expressed on myeloid cells 2; CB-TREM2- Cell Bound TREM2; IL-6 -interleukin 6; TNFα- tumor necrosis factor α; MCP-1 - monocyte chemoattractant protein-1. | | | | | | | | | | |  |

| Supplemental Table 5. Acute and chronic differentiation effect in Mo-Mф from CO- derived cells. | | | | | | | | | | |
| --- | --- | --- | --- | --- | --- | --- | --- | --- | --- | --- |
|  | **CO** | | | | | | | | | |
| Gene*/  Protein | **Acute Differentiation** | | | | | **Chronic Differentiation** | | | | |
|  | Friedman statistic | p-value | Dunn´s multiple comparison tests | | | Friedman statistic | p-value | Dunn´s multiple comparison tests | | |
|  |  |  | M1.vs M2 | M1.vs M0 | M2.vs M0 |  |  | M1.vs M2 | M1.vs M0 | M2.vs M0 |
| *trem2* | 9 | **0.0099** | **0.0081** | 0.4008 | 0.4008 | 10.75 | **0.0024** | **0.0035** | 0.1365 | 0.6339 |
| sTREM2 | 9.250 | **0.0080** | **0.0179** | **0.0373** | >0.9999 | 14.25 | **<0.0001** | **0.0733** | **0.0005** | 0.4008 |
| CB-TREM2 | 1.75 | 0.5306 | 0.9519 | 0.6339 | >0.9999 | 3 | 0.2851 | 0.4008 | 0.4008 | >0.9999 |
| *cd206* | 9 | **0.0099** | **0.0081** | 0.4008 | 0.4008 | 13 | **0.0003** | **0.0014** | 0.9519 | 0.0373 |
| IL-6 | 12.80 | **0.0005** | **0.0081** | **0.0081** | >0.9999 | 9.742 | **0.0046** | **0.0526** | **0.0121** | >0.9999 |
| TNF-α | 12.45 | **0.0005** | **0.0053** | **0.0121** | >0.9999 | 5.083 | 0.0847 | 0.2404 | 0.3125 | >0.9999 |
| mcp-1 | 4 | 0.1400 | 0.6339 | >0.9999 | 0.2404 | 6.083 | 0.0511 | 0.6339 | >0.9999 | 0.1008 |
| *qPCR products* (in italic)* and proteins calculated using Friedman’s ANOVA with Dunn´s multiple comparison tests for all measured markers in M1, M2, and M0 after acute and chronic differentiation. *TREM2 –* triggering receptor expressed on myeloid cells 2*; CD206 - cluster of differentiation 206 (mannose receptor);* sTREM2- soluble triggering receptor expressed on myeloid cells 2; CB-TREM2- Cell Bound TREM2; IL-6 -interleukin 6; TNFα- tumor necrosis factor α; MCP-1 - monocyte chemoattractant protein-1. | | | | | | | | | | |

| Supplemental Table 6. Differentiation effect on neuroinflammatory marker synthesis in Mo-Mф from APOE ε4 (+) - derived cells. | | | | | | | | | | |
| --- | --- | --- | --- | --- | --- | --- | --- | --- | --- | --- |
|  | **APOE ε4 (+)** | | | | | | | | | |
| Gene*/  Protein | **Acute Differentiation** | | | | | **Chronic Differentiation** | | | | |
|  | Friedman statistic | p-value | Dunn´s multiple comparison tests | | | Friedman statistic | p-value | Dunn´s multiple comparison tests | | |
|  |  |  | M1.vs M2 | M1.vs M0 | M2.vs M0 |  |  | M1.vs M2 | M1.vs M0 | M2.vs M0 |
| *trem2* | 7.548 | **0.0186** | **0.0260** | 0.1824 | >0.9999 | 9 | **0.0099** | **0.0081** | 0.4008 | 0.4008 |
| sTREM2 | 13.61 | **0.0001** | **0.0009** | 0.1365 | 0.3125 | 14.25 | **<0.0001** | **0.0733** | **0.0005** | 0.4008 |
| CB-TREM2 | 5.250 | **0.0789** | **0.0733** | >0.9999 | 0.4008 | 5.250 | 0.0789 | 0.4008 | 0.0733 | >0.9999 |
| *cd 206* | 6.750 | **0.0375** | 0.0733 | >0.9999 | 0.0733 | 7 | **0.0303** | **0.0373** | >0.9999 | 0.1365 |
| IL-6 | 12.97 | **0.0003** | **0.0022** | 0.0269 | >0.9999 | 9.250 | **0.0080** | **0.0179** | **0.0373** | >0.9999 |
| TNF-α | 13 | **0.0003** | **0.0014** | 0.0373 | 0.9519 | 6.5 | **0.0399** | 0.1365 | 0.1008 | >0.9999 |
| mcp-1 | 6.333 | **0.0384** | 0.1364 | >0.9999 | 0.2404 | 7.714 | **0.0103** | 0.7818 | 0.7818 | 0.0733 |
| *qPCR products* represented in italic and proteins calculated with Friedman’s ANOVA test with Dunn´s multiple comparison tests for all measured neuroinflammatory markers in M1, M2, and M0 in acute and chronic differentiation..TREM2 –* triggering receptor expressed on myeloid cells 2*; CD206 - cluster of differentiation 206 (mannose receptor); sTREM2- soluble* triggering receptor expressed on myeloid cells 2; *CB-TREM2- Cell Bound TREM2; IL-6 -interleukin 6; TNF*α*-* tumor necrosis factor α*; MCP-1 - monocyte chemoattractant protein-1.* | | | | | | | | | | |

| Supplemental Table 7. Differentiation effect on marker synthesis in Mo-Mф from APOE ε4 (-) - derived cells. | | | | | | | | | | |
| --- | --- | --- | --- | --- | --- | --- | --- | --- | --- | --- |
|  | **APOE ε4 (-)** | | | | | | | | | |
| *Gene**/  Protein | **Acute Differentiation** | | | | | **Chronic Differentiation** | | | | |
|  | Friedman statistic | p-value | Dunn´s multiple comparison tests | | | Friedman statistic | p-value | Dunn´s multiple comparison tests | | |
|  |  |  | M1.vs M2 | M1.vs M0 | M2.vs M0 |  |  | M1.vs M2 | M1.vs M0 | M2.vs M0 |
| *trem2** | 5.871 | 0.0609 | 0.0526 | 0.9519 | 0.5074 | 9 | **0.0099** | **0.0081** | 0.4008 | 0.4008 |
| sTREM2 | 7 | **0.0303** | >0.9999 | **0.0373** | 0.1365 | 12 | **0.0011** | **0.0081** | **0.0081** | >0.9999 |
| CB-TREM2 | 2 | 0.4097 | >0.9999 | >0.9999 | 0.5074 | 7 | **0.0303** | 0.1365 | 0.0373 | >0.9999 |
| *cd 206** | 6.250 | **0.0464** | 0.0373 | 0.6339 | 0.6339 | 14.25 | **<0.0001** | **0.0005** | 0.4008 | 0.0733 |
| IL-6 | 11.47 | **0.0012** | **0.0035** | 0.1365 | 0.6339 | 9.484 | **0.0058** | **0.0260** | **0.0260** | >0.9999 |
| TNF-a | 13.87 | **0.0001** | **0.0014** | **0.0373** | 0.9519 | 8.273 | **0.0123** | 0.0526 | 0.5074 | 0.9519 |
| mcp-1 | 10.40 | **0.0028** | 0.0733 | >0.9999 | 0.0081 | 10.57 | **0.0025** | **0.0179** | >0.9999 | 0.0373 |
| *qPCR products* represented in italic and proteins calculated with Friedman’s ANOVA test with Dunn´s multiple comparison tests for all measured neuroinflammatory markers in M1, M2, and M0 in acute and chronic differentiation.. TREM2 –* triggering receptor expressed on myeloid cells 2*; CD206 – cluster of differentiation 206 (mannose receptor);sTREM2- soluble* triggering receptor expressed on myeloid cells 2; *CB-TREM2- Cell Bound TREM2; IL-6 -interleukin 6; TNF*α*-* tumor necrosis factor α*; MCP-1 - monocyte chemoattractant protein-1.* | | | | | | | | | | |
